# Reproducibility discrepancies following reanalysis of raw data for a previously published study on diisononyl phthalate (DINP) in rats

**DOI:** 10.1016/j.dib.2017.05.043

**Published:** 2017-05-26

**Authors:** Min Chen, Rebecca Alyea, Peter Morfeld, Rainer Otter, Jessica Kemmerling, Christine Palermo

**Affiliations:** a ExxonMobil Biomedical Sciences Inc., 1545 Route 22 East, Annandale, NJ 08801, USA; b Institute for Occupational Epidemiology and Risk Assessment (IERA) of Evonik Industries, Rellinghauser Str. 1-11, 45128 Essen, Germany; c Institute and Policlinic for Occupational Medicine, Environmental Medicine and Prevention Research of Cologne University, Kerpener Strasse 62, 50937 Köln, Germany; d BASF SE, Carl-Bosch-Strasse 38, 67056 Ludwigshafen, Germany; e Evonik Performance Materials GmbH, Paul-Baumann-Strasse 1, 45772 Marl, Germany; f ExxonMobil Biomedical Sciences Inc., Hermeslaan 2, 1831 Machelen, Belgium

**Keywords:** Reproducibility, Reproductive toxicology, Statistical reanalysis, Diisononyl phthalate (DINP)

## Abstract

A 2011 publication by Boberg et al. entitled “Reproductive and behavioral effects of diisononyl phthalate (DINP) in perinatally exposed rats” [1] reported statistically significant changes in sperm parameters, testicular histopathology, anogenital distance and retained nipples in developing males. Using the statistical methods as reported by Boberg et al. (2011) [1], we reanalyzed the publically available raw data ([dataset] US EPA (United States Environmental Protection Agency), 2016) [2]. The output of our reanalysis and the discordances with the data as published in Boberg et al. (2011) [1] are highlighted herein. Further discussion of the basis for the replication discordances and the insufficiency of the Boberg et al. (2011) [1] response to address them can be found in a companion letter of correspondence (doi: 10.1016/j.reprotox.2017.03.013.; (Morfeld et al., 2011) [3]).

**Specifications Table**

**Table:** 

Subject area	Toxicology
More specific subject area	Reproducibility
Type of data	Tables, figures, text files, SAS (Statistical Analysis System) code
How data was acquired	Public database
Data format	Raw
Experimental factors	Statistical reanalysis of previously published results
Experimental features	Reproducibility discrepancies were identified following a reanalysis of the raw data from a previously published study according to the originally published statistical methods.
Data source location	*N/A*
Data accessibility	US Environmental Protection Agency public docket to accept public comments on IRIS assessment materials related to DINP
	Docket ID: EPA-HQ-ORD-2014-0637
	Supplemental Boberg data for HERO ID 806135 from email communication.EPA-Hq-ORD-2014-0637. https://www.regulations.gov/document?D=EPA-HQ-ORD-2014-0637-0014 Last accessed January 2017

**Value of the data**

• Corrected statistical data analysis of a published dataset [1].
• These data should be compared with the previously reported data by Boberg et al. [1], [4] taking into consideration the perspective offered in the companion letter of correspondence to this Data in Brief [3] to inform interpretation.
• Reanalysis of an existing data set adds value and confidence in targeted exploratory science.
• Reproducibility and accurate reporting of data are paramount when a particular dataset is being evaluated in the scientific and regulatory community.

## 1 Data

**Fig. 1:**
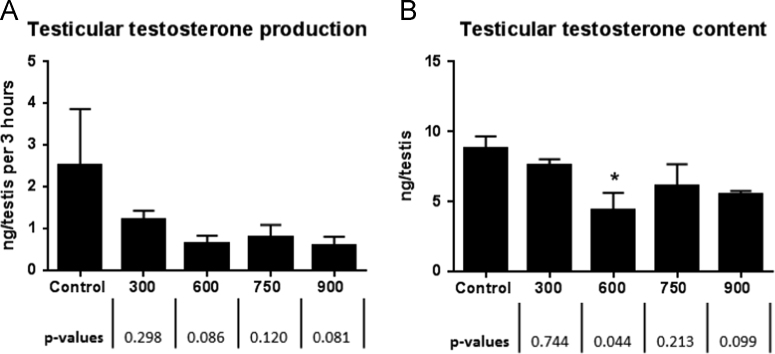
Reanalysis of male testosterone levels as reported in Boberg et al. [1] at GD 21 in rat fetuses following exposure to increasing concentrations of DINP. (A) Testicular testosterone production *ex vivo* (*n*=3–4 litters, 2 testes per litter), (B) testicular testosterone content (*n*=3–4 litters, 1–2 testes/litter). Mean±SEM (standard error of the mean), *p<0.05 compared to control. There is a discrepancy in the *y*-axis scaling between Fig. 2B herein and the comparable figure in Boberg et al. [1]. Statistically significant outcomes reported in Boberg et al. [1] were replicated.

**Fig. 2:**
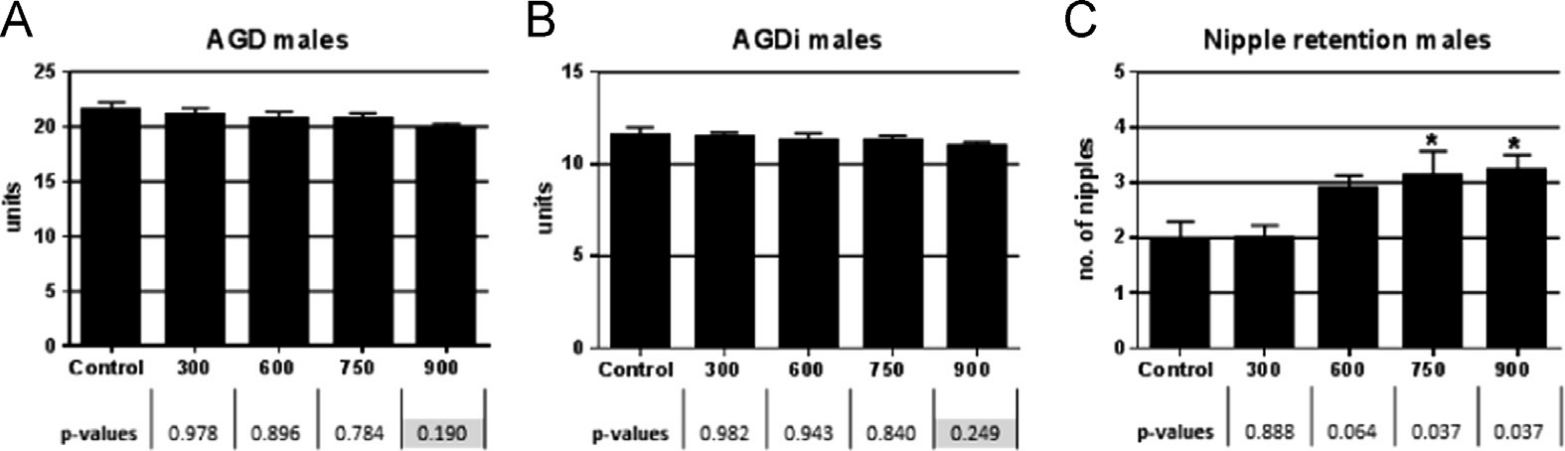
Reanalysis of the raw data in Boberg et al. [1] of male AGD, AGDi and number (no.) of nipples in male rats. Mean±SEM *p<0.05 compared to control. Gray highlighted *p*-values indicate where statistically significant outcomes of *p*<0.05 reported in Boberg et al. [1] were not replicated.

**Fig. 3:**
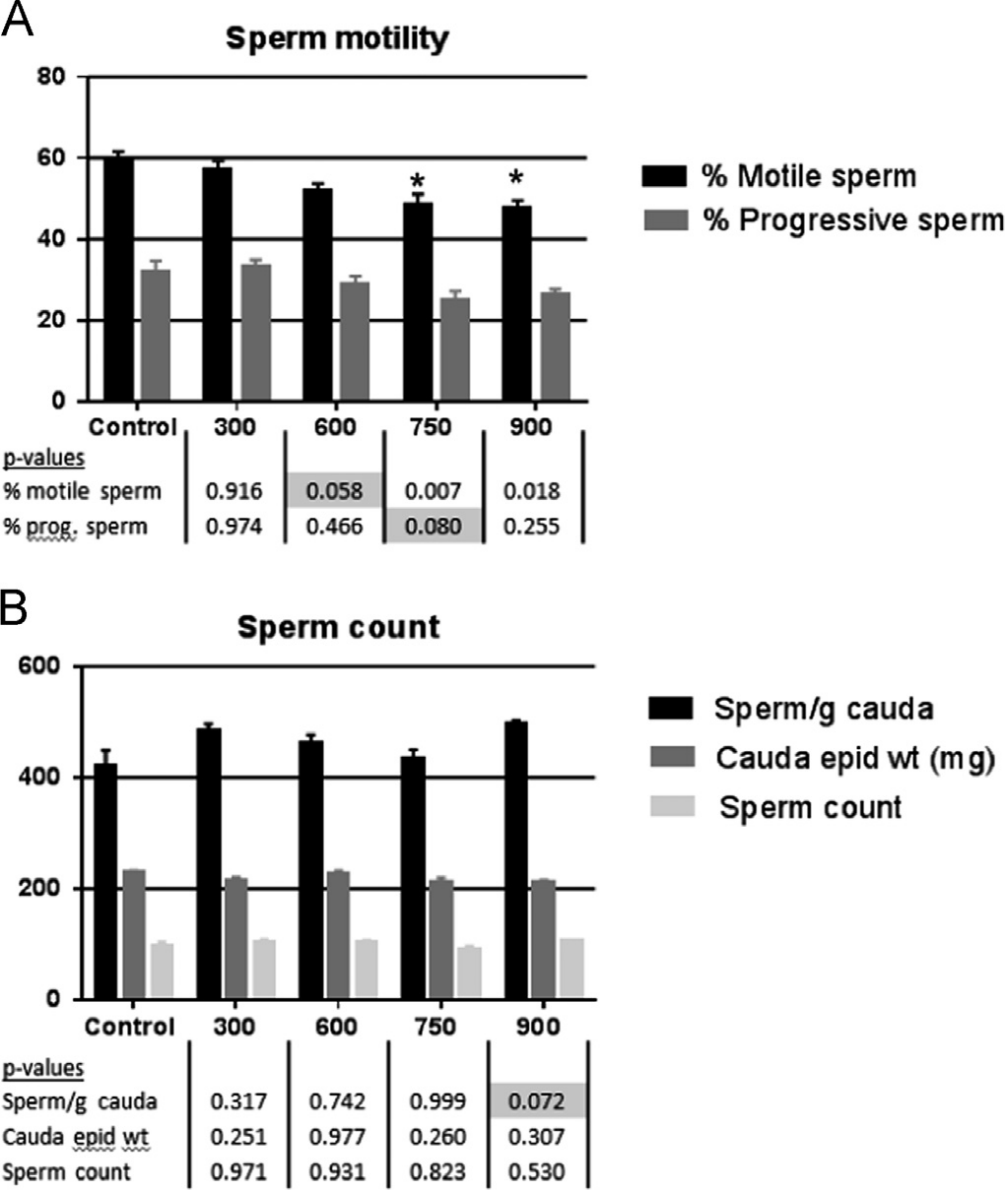
Reanalysis of the raw data in Boberg et al. [1] of male sperm motility and sperm count in 90 day old rats exposed to increasing concentrations of DINP from GD 7 to PND 17. Mean±SEM, *p<0.05 compared to control. Gray highlighted *p*-values indicate where statistically significant outcomes of *p*<0.05 reported in Boberg et al. [1] were not replicated. Cauda epid wt refers to Cauda epididymis weight. Refer to the companion correspondence to this publication [3] for discussion of concerns regarding experimental optimization for these endpoints.

The US EPA HERO database [2] has made publically available a portion of the raw data from a 2011 publication by Boberg et al. [1] on the reproductive and behavioral effects of diisononyl phthalate (DINP) in perinatally exposed rats. Upon reanalysis of the available raw data using the statistical methods as originally reported in the Materials and Methods section of Boberg et al. [1], we were unable to confirm the reported statistical significance for one or more DINP dose groups for testes histopathology outcomes (Table 1), male anogenital distance (AGD) measurements (Fig. 2; Table 2), percent progressive sperm, sperm/g cauda, and sperm motility (Fig. 3).The statistically significant outcomes for testicular testosterone (Fig. 1) and nipple retention (Fig. 2) were consistent with those reported in Boberg et al. [1]. However, the mean and standard deviations for nipples in males for the highest DINP dose group were not confirmed (Table 1); and the testicular content values reported in the raw data file [2] are substantially different from the testicular content values reflected by the y-axis in Figure 2B of Boberg et al. [1]. This reanalysis reports outcomes according to our understanding of the statistical methodology as originally reported by Boberg et al. [1]. Supplementary material contains the statistical reanalysis for the dataset (Fig. 1).

**Table 1:** Reanalysis of testis histology outcomes in rat fetuses (GD 21) exposed to increasing concentrations of DINP from GD 7 to 21. As described in Boberg et al. [1], one testis section was evaluated from 1 to 4 males per litter.

Table 1 lists percent animals affected, affected animals/total number of animals and (affected litters/total number of litters). Severity of histopathology finding was not reported in [1]. Results in **bold** are significantly different from controls in a one-sided Fisher׳s exact test (*p*≤0.05) corrected for multiple comparisons. *p*-values for affected animals /total number of animals and (affected litters/total number of litters) are also captured. *p*-values highlighted in gray indicate where statistically significant outcomes of *p*≤0.05 reported in Boberg et al. [1] were not replicated. As reported in Boberg at al. [1], the testis was damaged and testis histology could not be evaluated in one of the six animals evaluated in this group; however the presence of multinucleated gonocytes was noted.

**Table 2:** Reanalysis of birth weights, AGD, AGDi (PND1)and nipple retention (PND 13) for pups exposed to DINP from GD 7 to PND 17 (mean±Standard deviation).

Analysis performed as reported in Boberg et al. [1].

Gray highlights indicate replication discrepancies with Boberg et al. [1].

^a^Analyzed with body weight as a covariate.

^b^AGDi is defined as AGD divided by the cube root of the body weight.

^⁎^GEE with Rom [5] adjusted *p*-value<0.05.

## 2 Experimental design, materials and methods

The raw data were made publically available from the US EPA HERO database [2] in connection with the US EPA IRIS DINP review.

The statistical analysis was performed for all endpoints available in the raw data file [2] per the methodology described in Boberg et al. [1].

For testis histopathology, Table 1 in Boberg et al. [1] indicates one-sided Fisher׳s exact test, whereas the Materials and Methods indicates Fisher׳s exact test (one-sided) with *p*-value adjustments for multiple comparisons carried out by the ROM method [5]. Considering correction for multiple comparisons was included by Boberg et al. [1] in the statistical methods for AGD, sperm parameters, testosterone, and nipples, in this reanalysis we used Fisher׳s exact test (one-sided) against control group with *p*-value adjustments for multiple comparisons carried out by the ROM method [5].

When more than one pup from each litter was examined, statistical analyses was performed using litter as an independent, random and nested factor in ANOVA. Dunnett׳s test was performed to determine differences between treated and control group means. Data were examined to satisfy the assumption of normal distribution and homogeneity of variance for ANOVA test. These methods were applied to the raw data for testicular testosterone production *ex vivo*, testicular testosterone content, AGD, AGDi, and all sperm parameters. For AGD, the data analysis included body weight as a covariate in the analysis, to correct for the relationship between body size and AGD. AGDi was calculated by dividing AGD by the cubic root of the body weight.

For the number of nipples, Table 3 footnote c in Boberg et al. [1] indicates ANOVA followed by the Dunnett׳s test, whereas generalized linear models in combination with generalized estimating equations (GEE) were described in the Materials and Methods section. In this reanalysis, we used generalized linear models in combination with GEE. The number of nipples were analyzed by generalized linear models in combination with GEE in order to account for the nested litter correlation, with *p*-value adjustments carried out by the ROM method [5].

Statistical analyses were done using the SAS procedure PROC FREQ for Fisher׳s exact test, PROC MIXED for ANOVA followed by Dunnett׳s test, PROC GENMOD for GEE (SAS version 9.3, SAS Institute, Cary, NC, USA) and R version 3.2.5 scmamp package for Rom [5] adjusted *p*-value. The supplemental files to this publication contains the SAS code and reanalysis results.
