# Supplementary material for: Reproducibility discrepancies following reanalysis of raw data for a previously published study on diisononyl phthalate (DINP) in rats
Source: Data Brief. 2017 May 26;13:208–13. doi: 10.1016/j.dib.2017.05.043 (PMC5459566; doi:10.1016/j.dib.2017.05.043)
Supplement: Supplementary file 1 — Supplementary material [file mmc1.docx]

Conflict of interest statement

R. Otter reports personal fees from BASF SE, and J.Kemmerling reports personal fees from Evonik Performance Materials GmbH, outside the submitted work; C Palermo, R. Alyea, M. Chen, R. Otter, J. Kemmerling are employed by companies that manufacturer chemical products. .
