# Supplementary material for: Reproducibility discrepancies following reanalysis of raw data for a previously published study on diisononyl phthalate (DINP) in rats
Source: Data Brief. 2017 May 26;13:208–13. doi: 10.1016/j.dib.2017.05.043 (PMC5459566; doi:10.1016/j.dib.2017.05.043)
Supplement: Supplementary file 2 — Supplementary material [file mmc2.rtf]

Model Information	
Data Set	WORK.TEMP	
Dependent Variable	AG_male	
Covariance Structure	Variance Components	
Estimation Method	REML	
Residual Variance Method	Profile	
Fixed Effects SE Method	Model-Based	
Degrees of Freedom Method	Containment	


Class Level Information	
Class	Levels	Values	
Litter	43	4 7 8 11 12 16 19 20 21 24 26 28 29 30 31 32 33 35 36 37 38 39 40 47 48 56 59 60 61 62 63 64 67 68 69 70 71 72 73 74 76 78 80	
Group	5	1 2 3 4 5	


Dimensions	
Covariance Parameters	2	
Columns in X	7	
Columns in Z	43	
Subjects	1	
Max Obs Per Subject	435	


Number of Observations	
Number of Observations Read	435	
Number of Observations Used	202	
Number of Observations Not Used	233	


Covariance Parameter Estimates	
Cov Parm	Estimate	
Litter(Group)	2.2044	
Residual	2.6146	


Fit Statistics	
-2 Res Log Likelihood	824.5	
AIC (smaller is better)	828.5	
AICC (smaller is better)	828.6	
BIC (smaller is better)	832.1	


Type 3 Tests of Fixed Effects	
Effect	Num DF	Den DF	F Value	Pr > F	
Group	4	38	1.05	0.3958	
Birth_bw_male	1	158	7.53	0.0068	


Least Squares Means	
Effect	Group	Estimate	Standard Error	DF	t Value	Pr > |t|	
Group	1	21.3410	0.6320	38	33.77	<.0001	
Group	2	20.9743	0.6010	38	34.90	<.0001	
Group	3	20.7891	0.5262	38	39.50	<.0001	
Group	4	20.5848	0.5981	38	34.42	<.0001	
Group	5	19.7510	0.5507	38	35.86	<.0001	


Differences of Least Squares Means	
Effect	Group	Group	Estimate	Standard Error	DF	t Value	Pr > |t|	Adjustment	Adj P	
Group	2	1	-0.3667	0.8685	38	-0.42	0.6753	Dunnett-Hsu	0.9778	
Group	3	1	-0.5519	0.8228	38	-0.67	0.5065	Dunnett-Hsu	0.8959	
Group	4	1	-0.7562	0.8704	38	-0.87	0.3904	Dunnett-Hsu	0.7841	
Group	5	1	-1.5900	0.8414	38	-1.89	0.0664	Dunnett-Hsu	0.1900	

Model Information	
Data Set	WORK.TEMP	
Dependent Variable	AGI_male	
Covariance Structure	Variance Components	
Estimation Method	REML	
Residual Variance Method	Profile	
Fixed Effects SE Method	Model-Based	
Degrees of Freedom Method	Containment	


Class Level Information	
Class	Levels	Values	
Litter	46	4 7 8 11 12 16 19 20 21 24 25 26 28 29 30 31 32 33 35 36 37 38 39 40 43 47 48 55 56 59 60 61 62 63 64 67 68 69 70 71 72 73 74 76 78 80	
Group	5	1 2 3 4 5	


Dimensions	
Covariance Parameters	2	
Columns in X	6	
Columns in Z	46	
Subjects	1	
Max Obs Per Subject	435	


Number of Observations	
Number of Observations Read	435	
Number of Observations Used	202	
Number of Observations Not Used	233	


Covariance Parameter Estimates	
Cov Parm	Estimate	
Litter(Group)	0.6673	
Residual	0.7744	


Fit Statistics	
-2 Res Log Likelihood	585.9	
AIC (smaller is better)	589.9	
AICC (smaller is better)	589.9	
BIC (smaller is better)	593.5	


Type 3 Tests of Fixed Effects	
Effect	Num DF	Den DF	F Value	Pr > F	
Group	4	38	0.90	0.4760	


Least Squares Means	
Effect	Group	Estimate	Standard Error	DF	t Value	Pr > |t|	
Group	1	11.6254	0.3450	38	33.70	<.0001	
Group	2	11.4356	0.3289	38	34.77	<.0001	
Group	3	11.3748	0.2889	38	39.37	<.0001	
Group	4	11.2553	0.3282	38	34.29	<.0001	
Group	5	10.8283	0.3014	38	35.92	<.0001	


Differences of Least Squares Means	
Effect	Group	Group	Estimate	Standard Error	DF	t Value	Pr > |t|	Adjustment	Adj P	
Group	2	1	-0.1898	0.4766	38	-0.40	0.6927	Dunnett	0.9820	
Group	3	1	-0.2506	0.4500	38	-0.56	0.5809	Dunnett	0.9425	
Group	4	1	-0.3701	0.4762	38	-0.78	0.4418	Dunnett	0.8403	
Group	5	1	-0.7971	0.4581	38	-1.74	0.0900	Dunnett	0.2489	

Model Information	
Data Set	WORK.TEMP	
Dependent Variable	AG_fem1	
Covariance Structure	Variance Components	
Estimation Method	REML	
Residual Variance Method	Profile	
Fixed Effects SE Method	Model-Based	
Degrees of Freedom Method	Containment	


Class Level Information	
Class	Levels	Values	
Litter	45	4 7 8 11 12 16 19 20 21 24 25 26 28 29 30 31 32 33 35 36 37 38 39 40 43 47 48 55 56 59 60 61 62 63 64 67 68 69 70 71 72 73 74 76 78	
Group	5	1 2 3 4 5	


Dimensions	
Covariance Parameters	2	
Columns in X	7	
Columns in Z	45	
Subjects	1	
Max Obs Per Subject	435	


Number of Observations	
Number of Observations Read	435	
Number of Observations Used	227	
Number of Observations Not Used	208	


Covariance Parameter Estimates	
Cov Parm	Estimate	
Litter(Group)	0.6577	
Residual	0.6074	


Fit Statistics	
-2 Res Log Likelihood	609.9	
AIC (smaller is better)	613.9	
AICC (smaller is better)	614.0	
BIC (smaller is better)	617.5	


Type 3 Tests of Fixed Effects	
Effect	Num DF	Den DF	F Value	Pr > F	
Group	4	40	0.43	0.7853	
Birth_bw_fem1	1	181	25.01	<.0001	


Least Squares Means	
Effect	Group	Estimate	Standard Error	DF	t Value	Pr > |t|	
Group	1	11.0215	0.3164	40	34.84	<.0001	
Group	2	11.2987	0.2976	40	37.96	<.0001	
Group	3	11.1680	0.2931	40	38.11	<.0001	
Group	4	11.2368	0.2954	40	38.04	<.0001	
Group	5	10.8062	0.3006	40	35.95	<.0001	


Differences of Least Squares Means	
Effect	Group	Group	Estimate	Standard Error	DF	t Value	Pr > |t|	Adjustment	Adj P	
Group	2	1	0.2772	0.4342	40	0.64	0.5268	Dunnett-Hsu	0.9140	
Group	3	1	0.1464	0.4306	40	0.34	0.7356	Dunnett-Hsu	0.9904	
Group	4	1	0.2153	0.4329	40	0.50	0.6217	Dunnett-Hsu	0.9623	
Group	5	1	-0.2153	0.4369	40	-0.49	0.6248	Dunnett-Hsu	0.9635	

Model Information	
Data Set	WORK.TEMP	
Dependent Variable	AGI_female1	
Covariance Structure	Variance Components	
Estimation Method	REML	
Residual Variance Method	Profile	
Fixed Effects SE Method	Model-Based	
Degrees of Freedom Method	Containment	


Class Level Information	
Class	Levels	Values	
Litter	46	4 7 8 11 12 16 19 20 21 24 25 26 28 29 30 31 32 33 35 36 37 38 39 40 43 47 48 55 56 59 60 61 62 63 64 67 68 69 70 71 72 73 74 76 78 80	
Group	5	1 2 3 4 5	


Dimensions	
Covariance Parameters	2	
Columns in X	6	
Columns in Z	46	
Subjects	1	
Max Obs Per Subject	435	


Number of Observations	
Number of Observations Read	435	
Number of Observations Used	227	
Number of Observations Not Used	208	


Covariance Parameter Estimates	
Cov Parm	Estimate	
Litter(Group)	0.1983	
Residual	0.1868	


Fit Statistics	
-2 Res Log Likelihood	346.5	
AIC (smaller is better)	350.5	
AICC (smaller is better)	350.5	
BIC (smaller is better)	354.1	


Type 3 Tests of Fixed Effects	
Effect	Num DF	Den DF	F Value	Pr > F	
Group	4	40	0.45	0.7720	


Least Squares Means	
Effect	Group	Estimate	Standard Error	DF	t Value	Pr > |t|	
Group	1	6.1246	0.1738	40	35.23	<.0001	
Group	2	6.2754	0.1637	40	38.34	<.0001	
Group	3	6.2068	0.1608	40	38.59	<.0001	
Group	4	6.2359	0.1625	40	38.39	<.0001	
Group	5	5.9968	0.1652	40	36.31	<.0001	


Differences of Least Squares Means	
Effect	Group	Group	Estimate	Standard Error	DF	t Value	Pr > |t|	Adjustment	Adj P	
Group	2	1	0.1508	0.2388	40	0.63	0.5313	Dunnett	0.9169	
Group	3	1	0.08217	0.2368	40	0.35	0.7304	Dunnett	0.9897	
Group	4	1	0.1113	0.2379	40	0.47	0.6425	Dunnett	0.9695	
Group	5	1	-0.1278	0.2398	40	-0.53	0.5971	Dunnett	0.9523	

Model Information	
Data Set	WORK.TEMP1	
Dependent Variable	Motile_sperm	
Covariance Structure	Variance Components	
Estimation Method	REML	
Residual Variance Method	Profile	
Fixed Effects SE Method	Model-Based	
Degrees of Freedom Method	Containment	


Class Level Information	
Class	Levels	Values	
Litter	37	4 7 8 11 12 16 19 21 24 28 29 30 31 32 33 35 37 38 40 47 48 56 59 60 61 62 64 67 68 69 70 71 72 73 74 76 78	
Group	5	Control DINP 300 DINP 600 DINP 750 DINP 900	


Dimensions	
Covariance Parameters	2	
Columns in X	6	
Columns in Z	37	
Subjects	1	
Max Obs Per Subject	75	


Number of Observations	
Number of Observations Read	75	
Number of Observations Used	75	
Number of Observations Not Used	0	


Covariance Parameter Estimates	
Cov Parm	Estimate	
Litter(Group)	2.8582	
Residual	74.5369	


Fit Statistics	
-2 Res Log Likelihood	516.3	
AIC (smaller is better)	520.3	
AICC (smaller is better)	520.5	
BIC (smaller is better)	523.5	


Type 3 Tests of Fixed Effects	
Effect	Num DF	Den DF	F Value	Pr > F	
Group	4	32	4.50	0.0054	


Least Squares Means	
Effect	Group	Estimate	Standard Error	DF	t Value	Pr > |t|	
Group	Control	59.2544	2.2691	32	26.11	<.0001	
Group	DINP 300	57.1723	2.2637	32	25.26	<.0001	
Group	DINP 600	51.2706	2.2293	32	23.00	<.0001	
Group	DINP 750	47.9445	2.4874	32	19.27	<.0001	
Group	DINP 900	49.3187	2.4114	32	20.45	<.0001	


Differences of Least Squares Means	
Effect	Group	Group	Estimate	Standard Error	DF	t Value	Pr > |t|	Adjustment	Adj P	
Group	DINP 300	Control	-2.0821	3.2051	32	-0.65	0.5206	Dunnett	0.9160	
Group	DINP 600	Control	-7.9838	3.1810	32	-2.51	0.0173	Dunnett	0.0577	
Group	DINP 750	Control	-11.3099	3.3669	32	-3.36	0.0020	Dunnett	0.0074	
Group	DINP 900	Control	-9.9357	3.3111	32	-3.00	0.0052	Dunnett	0.0182	

Model Information	
Data Set	WORK.TEMP1	
Dependent Variable	Progressive_sperm	
Covariance Structure	Variance Components	
Estimation Method	REML	
Residual Variance Method	Profile	
Fixed Effects SE Method	Model-Based	
Degrees of Freedom Method	Containment	


Class Level Information	
Class	Levels	Values	
Litter	37	4 7 8 11 12 16 19 21 24 28 29 30 31 32 33 35 37 38 40 47 48 56 59 60 61 62 64 67 68 69 70 71 72 73 74 76 78	
Group	5	Control DINP 300 DINP 600 DINP 750 DINP 900	


Dimensions	
Covariance Parameters	2	
Columns in X	6	
Columns in Z	37	
Subjects	1	
Max Obs Per Subject	75	


Number of Observations	
Number of Observations Read	75	
Number of Observations Used	75	
Number of Observations Not Used	0	


Covariance Parameter Estimates	
Cov Parm	Estimate	
Litter(Group)	15.3060	
Residual	32.1594	


Fit Statistics	
-2 Res Log Likelihood	475.8	
AIC (smaller is better)	479.8	
AICC (smaller is better)	480.0	
BIC (smaller is better)	483.0	


Type 3 Tests of Fixed Effects	
Effect	Num DF	Den DF	F Value	Pr > F	
Group	4	32	2.84	0.0403	


Least Squares Means	
Effect	Group	Estimate	Standard Error	DF	t Value	Pr > |t|	
Group	Control	32.0618	2.1446	32	14.95	<.0001	
Group	DINP 300	33.4191	2.0957	32	15.95	<.0001	
Group	DINP 600	28.1622	1.9054	32	14.78	<.0001	
Group	DINP 750	24.8481	2.1926	32	11.33	<.0001	
Group	DINP 900	26.7127	2.1804	32	12.25	<.0001	


Differences of Least Squares Means	
Effect	Group	Group	Estimate	Standard Error	DF	t Value	Pr > |t|	Adjustment	Adj P	
Group	DINP 300	Control	1.3573	2.9986	32	0.45	0.6539	Dunnett	0.9738	
Group	DINP 600	Control	-3.8996	2.8688	32	-1.36	0.1835	Dunnett	0.4658	
Group	DINP 750	Control	-7.2137	3.0671	32	-2.35	0.0250	Dunnett	0.0798	
Group	DINP 900	Control	-5.3491	3.0584	32	-1.75	0.0899	Dunnett	0.2551	

Model Information	
Data Set	WORK.TEMP1	
Dependent Variable	Sperm_g	
Covariance Structure	Variance Components	
Estimation Method	REML	
Residual Variance Method	Profile	
Fixed Effects SE Method	Model-Based	
Degrees of Freedom Method	Containment	


Class Level Information	
Class	Levels	Values	
Litter	37	4 7 8 11 12 16 19 21 24 28 29 30 31 32 33 35 37 38 40 47 48 56 59 60 61 62 64 67 68 69 70 71 72 73 74 76 78	
Group	5	Control DINP 300 DINP 600 DINP 750 DINP 900	


Dimensions	
Covariance Parameters	2	
Columns in X	6	
Columns in Z	37	
Subjects	1	
Max Obs Per Subject	75	


Number of Observations	
Number of Observations Read	75	
Number of Observations Used	75	
Number of Observations Not Used	0	


Covariance Parameter Estimates	
Cov Parm	Estimate	
Litter(Group)	0	
Residual	6442.07	


Fit Statistics	
-2 Res Log Likelihood	826.1	
AIC (smaller is better)	828.1	
AICC (smaller is better)	828.2	
BIC (smaller is better)	829.7	


Type 3 Tests of Fixed Effects	
Effect	Num DF	Den DF	F Value	Pr > F	
Group	4	32	2.24	0.0861	


Least Squares Means	
Effect	Group	Estimate	Standard Error	DF	t Value	Pr > |t|	
Group	Control	428.44	20.0656	32	21.35	<.0001	
Group	DINP 300	474.78	20.0656	32	23.66	<.0001	
Group	DINP 600	455.85	20.0656	32	22.72	<.0001	
Group	DINP 750	423.44	22.2608	32	19.02	<.0001	
Group	DINP 900	499.34	21.4511	32	23.28	<.0001	


Differences of Least Squares Means	
Effect	Group	Group	Estimate	Standard Error	DF	t Value	Pr > |t|	Adjustment	Adj P	
Group	DINP 300	Control	46.3375	28.3771	32	1.63	0.1123	Dunnett	0.3166	
Group	DINP 600	Control	27.4062	28.3771	32	0.97	0.3414	Dunnett	0.7422	
Group	DINP 750	Control	-5.0053	29.9695	32	-0.17	0.8684	Dunnett	0.9994	
Group	DINP 900	Control	70.8920	29.3731	32	2.41	0.0217	Dunnett	0.0715	

Model Information	
Data Set	WORK.TEMP1	
Dependent Variable	Cauda_mg	
Covariance Structure	Variance Components	
Estimation Method	REML	
Residual Variance Method	Profile	
Fixed Effects SE Method	Model-Based	
Degrees of Freedom Method	Containment	


Class Level Information	
Class	Levels	Values	
Litter	37	4 7 8 11 12 16 19 21 24 28 29 30 31 32 33 35 37 38 40 47 48 56 59 60 61 62 64 67 68 69 70 71 72 73 74 76 78	
Group	5	Control DINP 300 DINP 600 DINP 750 DINP 900	


Dimensions	
Covariance Parameters	2	
Columns in X	6	
Columns in Z	37	
Subjects	1	
Max Obs Per Subject	75	


Number of Observations	
Number of Observations Read	75	
Number of Observations Used	75	
Number of Observations Not Used	0	


Covariance Parameter Estimates	
Cov Parm	Estimate	
Litter(Group)	82.9449	
Residual	455.47	


Fit Statistics	
-2 Res Log Likelihood	650.4	
AIC (smaller is better)	654.4	
AICC (smaller is better)	654.5	
BIC (smaller is better)	657.6	


Type 3 Tests of Fixed Effects	
Effect	Num DF	Den DF	F Value	Pr > F	
Group	4	32	1.45	0.2397	


Least Squares Means	
Effect	Group	Estimate	Standard Error	DF	t Value	Pr > |t|	
Group	Control	229.31	6.5267	32	35.13	<.0001	
Group	DINP 300	213.11	6.4588	32	32.99	<.0001	
Group	DINP 600	225.36	6.1151	32	36.85	<.0001	
Group	DINP 750	212.70	6.9256	32	30.71	<.0001	
Group	DINP 900	213.80	6.8090	32	31.40	<.0001	


Differences of Least Squares Means	
Effect	Group	Group	Estimate	Standard Error	DF	t Value	Pr > |t|	Adjustment	Adj P	
Group	DINP 300	Control	-16.2001	9.1823	32	-1.76	0.0872	Dunnett	0.2512	
Group	DINP 600	Control	-3.9511	8.9438	32	-0.44	0.6616	Dunnett	0.9767	
Group	DINP 750	Control	-16.6049	9.5164	32	-1.74	0.0906	Dunnett	0.2597	
Group	DINP 900	Control	-15.5038	9.4318	32	-1.64	0.1100	Dunnett	0.3074	

Model Information	
Data Set	WORK.TEMP1	
Dependent Variable	sperm_count	
Covariance Structure	Variance Components	
Estimation Method	REML	
Residual Variance Method	Profile	
Fixed Effects SE Method	Model-Based	
Degrees of Freedom Method	Containment	


Class Level Information	
Class	Levels	Values	
Litter	37	4 7 8 11 12 16 19 21 24 28 29 30 31 32 33 35 37 38 40 47 48 56 59 60 61 62 64 67 68 69 70 71 72 73 74 76 78	
Group	5	Control DINP 300 DINP 600 DINP 750 DINP 900	


Dimensions	
Covariance Parameters	2	
Columns in X	6	
Columns in Z	37	
Subjects	1	
Max Obs Per Subject	75	


Number of Observations	
Number of Observations Read	75	
Number of Observations Used	75	
Number of Observations Not Used	0	


Covariance Parameter Estimates	
Cov Parm	Estimate	
Litter(Group)	0	
Residual	387.86	


Fit Statistics	
-2 Res Log Likelihood	629.4	
AIC (smaller is better)	631.4	
AICC (smaller is better)	631.5	
BIC (smaller is better)	633.0	


Type 3 Tests of Fixed Effects	
Effect	Num DF	Den DF	F Value	Pr > F	
Group	4	32	1.13	0.3583	


Least Squares Means	
Effect	Group	Estimate	Standard Error	DF	t Value	Pr > |t|	
Group	Control	97.8394	4.9236	32	19.87	<.0001	
Group	DINP 300	101.14	4.9236	32	20.54	<.0001	
Group	DINP 600	102.10	4.9236	32	20.74	<.0001	
Group	DINP 750	91.6813	5.4622	32	16.78	<.0001	
Group	DINP 900	107.04	5.2635	32	20.34	<.0001	


Differences of Least Squares Means	
Effect	Group	Group	Estimate	Standard Error	DF	t Value	Pr > |t|	Adjustment	Adj P	
Group	DINP 300	Control	3.3036	6.9630	32	0.47	0.6384	Dunnett	0.9710	
Group	DINP 600	Control	4.2619	6.9630	32	0.61	0.5448	Dunnett	0.9312	
Group	DINP 750	Control	-6.1580	7.3537	32	-0.84	0.4086	Dunnett	0.8225	
Group	DINP 900	Control	9.1993	7.2073	32	1.28	0.2110	Dunnett	0.5300	

Model Information	
Data Set	WORK.TEMP2	
Dependent Variable	ng_per_testis	
Covariance Structure	Variance Components	
Estimation Method	REML	
Residual Variance Method	Profile	
Fixed Effects SE Method	Model-Based	
Degrees of Freedom Method	Containment	


Class Level Information	
Class	Levels	Values	
litter	17	1 5 6 9 10 14 15 17 18 41 42 45 46 51 52 53 58	
group	5	1 2 3 4 5	


Dimensions	
Covariance Parameters	2	
Columns in X	6	
Columns in Z	17	
Subjects	1	
Max Obs Per Subject	29	


Number of Observations	
Number of Observations Read	29	
Number of Observations Used	29	
Number of Observations Not Used	0	


Covariance Parameter Estimates	
Cov Parm	Estimate	
litter(group)	0.5039	
Residual	4.6160	


Fit Statistics	
-2 Res Log Likelihood	115.6	
AIC (smaller is better)	119.6	
AICC (smaller is better)	120.1	
BIC (smaller is better)	121.2	


Type 3 Tests of Fixed Effects	
Effect	Num DF	Den DF	F Value	Pr > F	
group	4	12	2.72	0.0803	


Least Squares Means	
Effect	group	Estimate	Standard Error	DF	t Value	Pr > |t|	
group	1	8.7864	0.9681	12	9.08	<.0001	
group	2	7.4810	0.9526	12	7.85	<.0001	
group	3	4.8770	0.9526	12	5.12	0.0003	
group	4	6.0020	1.0503	12	5.71	<.0001	
group	5	5.4768	0.9681	12	5.66	0.0001	


Differences of Least Squares Means	
Effect	group	_group	Estimate	Standard Error	DF	t Value	Pr > |t|	Adjustment	Adj P	
group	2	1	-1.3054	1.3582	12	-0.96	0.3555	Dunnett	0.7439	
group	3	1	-3.9094	1.3582	12	-2.88	0.0139	Dunnett	0.0443	
group	4	1	-2.7844	1.4284	12	-1.95	0.0750	Dunnett	0.2129	
group	5	1	-3.3096	1.3691	12	-2.42	0.0325	Dunnett	0.0988	

Model Information	
Data Set	WORK.TEMP3	
Dependent Variable	Testicular_testosterone_producti	
Covariance Structure	Variance Components	
Estimation Method	REML	
Residual Variance Method	Profile	
Fixed Effects SE Method	Model-Based	
Degrees of Freedom Method	Containment	


Class Level Information	
Class	Levels	Values	
litter	19	1 5 6 9 10 14 15 17 18 41 42 45 46 51 52 53 54 57 58	
group	5	1 2 3 4 5	


Dimensions	
Covariance Parameters	2	
Columns in X	6	
Columns in Z	19	
Subjects	1	
Max Obs Per Subject	38	


Number of Observations	
Number of Observations Read	38	
Number of Observations Used	37	
Number of Observations Not Used	1	


Covariance Parameter Estimates	
Cov Parm	Estimate	
litter(group)	0	
Residual	2.0224	


Fit Statistics	
-2 Res Log Likelihood	123.3	
AIC (smaller is better)	125.3	
AICC (smaller is better)	125.5	
BIC (smaller is better)	126.3	


Type 3 Tests of Fixed Effects	
Effect	Num DF	Den DF	F Value	Pr > F	
group	4	14	2.04	0.1439	


Least Squares Means	
Effect	group	Estimate	Standard Error	DF	t Value	Pr > |t|	
group	1	2.5067	0.5806	14	4.32	0.0007	
group	2	1.2163	0.5028	14	2.42	0.0298	
group	3	0.6425	0.5028	14	1.28	0.2221	
group	4	0.7850	0.5028	14	1.56	0.1408	
group	5	0.5586	0.5375	14	1.04	0.3163	


Differences of Least Squares Means	
Effect	group	_group	Estimate	Standard Error	DF	t Value	Pr > |t|	Adjustment	Adj P	
group	2	1	-1.2904	0.7680	14	-1.68	0.1151	Dunnett	0.2980	
group	3	1	-1.8642	0.7680	14	-2.43	0.0293	Dunnett	0.0863	
group	4	1	-1.7217	0.7680	14	-2.24	0.0417	Dunnett	0.1197	
group	5	1	-1.9481	0.7912	14	-2.46	0.0274	Dunnett	0.0810	

Model Information	
Data Set	WORK.TEMP1	
Dependent Variable	AP_vel	
Covariance Structure	Variance Components	
Estimation Method	REML	
Residual Variance Method	Profile	
Fixed Effects SE Method	Model-Based	
Degrees of Freedom Method	Containment	


Class Level Information	
Class	Levels	Values	
Litter	37	4 7 8 11 12 16 19 21 24 28 29 30 31 32 33 35 37 38 40 47 48 56 59 60 61 62 64 67 68 69 70 71 72 73 74 76 78	
Group	5	Control DINP 300 DINP 600 DINP 750 DINP 900	


Dimensions	
Covariance Parameters	2	
Columns in X	6	
Columns in Z	37	
Subjects	1	
Max Obs Per Subject	75	


Number of Observations	
Number of Observations Read	75	
Number of Observations Used	75	
Number of Observations Not Used	0	


Covariance Parameter Estimates	
Cov Parm	Estimate	
Litter(Group)	69.1503	
Residual	73.9080	


Fit Statistics	
-2 Res Log Likelihood	545.9	
AIC (smaller is better)	549.9	
AICC (smaller is better)	550.1	
BIC (smaller is better)	553.1	


Type 3 Tests of Fixed Effects	
Effect	Num DF	Den DF	F Value	Pr > F	
Group	4	32	0.80	0.5360	


Least Squares Means	
Effect	Group	Estimate	Standard Error	DF	t Value	Pr > |t|	
Group	Control	164.64	4.0324	32	40.83	<.0001	
Group	DINP 300	162.26	3.8909	32	41.70	<.0001	
Group	DINP 600	163.66	3.4399	32	47.58	<.0001	
Group	DINP 750	155.61	4.0051	32	38.85	<.0001	
Group	DINP 900	161.96	4.0038	32	40.45	<.0001	


Differences of Least Squares Means	
Effect	Group	Group	Estimate	Standard Error	DF	t Value	Pr > |t|	Adjustment	Adj P	
Group	DINP 300	Control	-2.3737	5.6035	32	-0.42	0.6747	Dunnett	0.9787	
Group	DINP 600	Control	-0.9716	5.3003	32	-0.18	0.8557	Dunnett	0.9991	
Group	DINP 750	Control	-9.0207	5.6834	32	-1.59	0.1223	Dunnett	0.3297	
Group	DINP 900	Control	-2.6761	5.6825	32	-0.47	0.6409	Dunnett	0.9690	

Model Information	
Data Set	WORK.TEMP1	
Dependent Variable	CL_vel	
Covariance Structure	Variance Components	
Estimation Method	REML	
Residual Variance Method	Profile	
Fixed Effects SE Method	Model-Based	
Degrees of Freedom Method	Containment	


Class Level Information	
Class	Levels	Values	
Litter	37	4 7 8 11 12 16 19 21 24 28 29 30 31 32 33 35 37 38 40 47 48 56 59 60 61 62 64 67 68 69 70 71 72 73 74 76 78	
Group	5	Control DINP 300 DINP 600 DINP 750 DINP 900	


Dimensions	
Covariance Parameters	2	
Columns in X	6	
Columns in Z	37	
Subjects	1	
Max Obs Per Subject	75	


Number of Observations	
Number of Observations Read	75	
Number of Observations Used	75	
Number of Observations Not Used	0	


Covariance Parameter Estimates	
Cov Parm	Estimate	
Litter(Group)	357.44	
Residual	218.61	


Fit Statistics	
-2 Res Log Likelihood	634.1	
AIC (smaller is better)	638.1	
AICC (smaller is better)	638.3	
BIC (smaller is better)	641.3	


Type 3 Tests of Fixed Effects	
Effect	Num DF	Den DF	F Value	Pr > F	
Group	4	32	0.70	0.5951	


Least Squares Means	
Effect	Group	Estimate	Standard Error	DF	t Value	Pr > |t|	
Group	Control	331.45	8.5810	32	38.63	<.0001	
Group	DINP 300	334.03	8.1895	32	40.79	<.0001	
Group	DINP 600	332.36	7.1069	32	46.77	<.0001	
Group	DINP 750	324.20	8.3432	32	38.86	<.0001	
Group	DINP 900	343.73	8.3559	32	41.14	<.0001	


Differences of Least Squares Means	
Effect	Group	Group	Estimate	Standard Error	DF	t Value	Pr > |t|	Adjustment	Adj P	
Group	DINP 300	Control	2.5790	11.8618	32	0.22	0.8293	Dunnett	0.9982	
Group	DINP 600	Control	0.9065	11.1419	32	0.08	0.9357	Dunnett	1.0000	
Group	DINP 750	Control	-7.2469	11.9684	32	-0.61	0.5491	Dunnett	0.9264	
Group	DINP 900	Control	12.2774	11.9773	32	1.03	0.3130	Dunnett	0.6850	

Model Information	
Data Set	WORK.TEMP1	
Dependent Variable	SL_vel	
Covariance Structure	Variance Components	
Estimation Method	REML	
Residual Variance Method	Profile	
Fixed Effects SE Method	Model-Based	
Degrees of Freedom Method	Containment	


Class Level Information	
Class	Levels	Values	
Litter	37	4 7 8 11 12 16 19 21 24 28 29 30 31 32 33 35 37 38 40 47 48 56 59 60 61 62 64 67 68 69 70 71 72 73 74 76 78	
Group	5	Control DINP 300 DINP 600 DINP 750 DINP 900	


Dimensions	
Covariance Parameters	2	
Columns in X	6	
Columns in Z	37	
Subjects	1	
Max Obs Per Subject	75	


Number of Observations	
Number of Observations Read	75	
Number of Observations Used	75	
Number of Observations Not Used	0	


Covariance Parameter Estimates	
Cov Parm	Estimate	
Litter(Group)	50.3895	
Residual	56.7048	


Fit Statistics	
-2 Res Log Likelihood	526.3	
AIC (smaller is better)	530.3	
AICC (smaller is better)	530.5	
BIC (smaller is better)	533.6	


Type 3 Tests of Fixed Effects	
Effect	Num DF	Den DF	F Value	Pr > F	
Group	4	32	0.36	0.8380	


Least Squares Means	
Effect	Group	Estimate	Standard Error	DF	t Value	Pr > |t|	
Group	Control	98.9799	3.4684	32	28.54	<.0001	
Group	DINP 300	98.5035	3.3501	32	29.40	<.0001	
Group	DINP 600	98.6739	2.9676	32	33.25	<.0001	
Group	DINP 750	94.7745	3.4523	32	27.45	<.0001	
Group	DINP 900	95.3721	3.4502	32	27.64	<.0001	


Differences of Least Squares Means	
Effect	Group	Group	Estimate	Standard Error	DF	t Value	Pr > |t|	Adjustment	Adj P	
Group	DINP 300	Control	-0.4764	4.8221	32	-0.10	0.9219	Dunnett	0.9999	
Group	DINP 600	Control	-0.3060	4.5647	32	-0.07	0.9470	Dunnett	1.0000	
Group	DINP 750	Control	-4.2054	4.8937	32	-0.86	0.3965	Dunnett	0.7980	
Group	DINP 900	Control	-3.6078	4.8922	32	-0.74	0.4662	Dunnett	0.8680	

Model Information	
Data Set	WORK.TEMP1	
Dependent Variable	STR	
Covariance Structure	Variance Components	
Estimation Method	REML	
Residual Variance Method	Profile	
Fixed Effects SE Method	Model-Based	
Degrees of Freedom Method	Containment	


Class Level Information	
Class	Levels	Values	
Litter	37	4 7 8 11 12 16 19 21 24 28 29 30 31 32 33 35 37 38 40 47 48 56 59 60 61 62 64 67 68 69 70 71 72 73 74 76 78	
Group	5	Control DINP 300 DINP 600 DINP 750 DINP 900	


Dimensions	
Covariance Parameters	2	
Columns in X	6	
Columns in Z	37	
Subjects	1	
Max Obs Per Subject	75	


Number of Observations	
Number of Observations Read	75	
Number of Observations Used	75	
Number of Observations Not Used	0	


Covariance Parameter Estimates	
Cov Parm	Estimate	
Litter(Group)	1.3742	
Residual	8.2767	


Fit Statistics	
-2 Res Log Likelihood	369.1	
AIC (smaller is better)	373.1	
AICC (smaller is better)	373.2	
BIC (smaller is better)	376.3	


Type 3 Tests of Fixed Effects	
Effect	Num DF	Den DF	F Value	Pr > F	
Group	4	32	0.35	0.8421	


Least Squares Means	
Effect	Group	Estimate	Standard Error	DF	t Value	Pr > |t|	
Group	Control	60.2728	0.8669	32	69.53	<.0001	
Group	DINP 300	60.9621	0.8586	32	71.00	<.0001	
Group	DINP 600	60.5344	0.8157	32	74.21	<.0001	
Group	DINP 750	60.7205	0.9226	32	65.81	<.0001	
Group	DINP 900	59.5862	0.9061	32	65.76	<.0001	


Differences of Least Squares Means	
Effect	Group	Group	Estimate	Standard Error	DF	t Value	Pr > |t|	Adjustment	Adj P	
Group	DINP 300	Control	0.6892	1.2202	32	0.56	0.5761	Dunnett	0.9456	
Group	DINP 600	Control	0.2615	1.1903	32	0.22	0.8275	Dunnett	0.9983	
Group	DINP 750	Control	0.4477	1.2660	32	0.35	0.7259	Dunnett	0.9897	
Group	DINP 900	Control	-0.6866	1.2540	32	-0.55	0.5878	Dunnett	0.9510	

Model Information	
Data Set	WORK.TEMP	
Dependent Variable	Nipples_male	
Covariance Structure	Variance Components	
Estimation Method	REML	
Residual Variance Method	Profile	
Fixed Effects SE Method	Model-Based	
Degrees of Freedom Method	Containment	


Class Level Information	
Class	Levels	Values	
Litter	46	4 7 8 11 12 16 19 20 21 24 25 26 28 29 30 31 32 33 35 36 37 38 39 40 43 47 48 55 56 59 60 61 62 63 64 67 68 69 70 71 72 73 74 76 78 80	
Group	5	1 2 3 4 5	


Dimensions	
Covariance Parameters	2	
Columns in X	6	
Columns in Z	46	
Subjects	1	
Max Obs Per Subject	435	


Number of Observations	
Number of Observations Read	435	
Number of Observations Used	196	
Number of Observations Not Used	239	


Covariance Parameter Estimates	
Cov Parm	Estimate	
Litter(Group)	0.4393	
Residual	1.1812	


Fit Statistics	
-2 Res Log Likelihood	628.2	
AIC (smaller is better)	632.2	
AICC (smaller is better)	632.3	
BIC (smaller is better)	635.9	


Type 3 Tests of Fixed Effects	
Effect	Num DF	Den DF	F Value	Pr > F	
Group	4	38	4.21	0.0064	


Least Squares Means	
Effect	Group	Estimate	Standard Error	DF	t Value	Pr > |t|	
Group	1	2.0103	0.3126	38	6.43	<.0001	
Group	2	1.9688	0.3027	38	6.50	<.0001	
Group	3	2.8813	0.2633	38	10.94	<.0001	
Group	4	3.2537	0.3019	38	10.78	<.0001	
Group	5	3.1317	0.2832	38	11.06	<.0001	


Differences of Least Squares Means	
Effect	Group	Group	Estimate	Standard Error	DF	t Value	Pr > |t|	Adjustment	Adj P	
Group	2	1	-0.04152	0.4352	38	-0.10	0.9245	Dunnett	0.9999	
Group	3	1	0.8710	0.4087	38	2.13	0.0396	Dunnett	0.1201	
Group	4	1	1.2434	0.4346	38	2.86	0.0068	Dunnett	0.0231	
Group	5	1	1.1214	0.4218	38	2.66	0.0114	Dunnett	0.0376	

Model Information	
Data Set	WORK.TEMP	
Dependent Variable	Nipples_female1	
Covariance Structure	Variance Components	
Estimation Method	REML	
Residual Variance Method	Profile	
Fixed Effects SE Method	Model-Based	
Degrees of Freedom Method	Containment	


Class Level Information	
Class	Levels	Values	
Litter	46	4 7 8 11 12 16 19 20 21 24 25 26 28 29 30 31 32 33 35 36 37 38 39 40 43 47 48 55 56 59 60 61 62 63 64 67 68 69 70 71 72 73 74 76 78 80	
Group	5	1 2 3 4 5	


Dimensions	
Covariance Parameters	2	
Columns in X	6	
Columns in Z	46	
Subjects	1	
Max Obs Per Subject	435	


Number of Observations	
Number of Observations Read	435	
Number of Observations Used	222	
Number of Observations Not Used	213	


Covariance Parameter Estimates	
Cov Parm	Estimate	
Litter(Group)	0	
Residual	0.2226	


Fit Statistics	
-2 Res Log Likelihood	308.7	
AIC (smaller is better)	310.7	
AICC (smaller is better)	310.7	
BIC (smaller is better)	312.6	


Type 3 Tests of Fixed Effects	
Effect	Num DF	Den DF	F Value	Pr > F	
Group	4	40	0.54	0.7062	


Least Squares Means	
Effect	Group	Estimate	Standard Error	DF	t Value	Pr > |t|	
Group	1	12.2857	0.07280	40	168.77	<.0001	
Group	2	12.2750	0.07459	40	164.56	<.0001	
Group	3	12.3111	0.07033	40	175.05	<.0001	
Group	4	12.2500	0.06809	40	179.90	<.0001	
Group	5	12.3830	0.06881	40	179.95	<.0001	


Differences of Least Squares Means	
Effect	Group	Group	Estimate	Standard Error	DF	t Value	Pr > |t|	Adjustment	Adj P	
Group	2	1	-0.01071	0.1042	40	-0.10	0.9186	Dunnett	0.9999	
Group	3	1	0.02540	0.1012	40	0.25	0.8032	Dunnett	0.9971	
Group	4	1	-0.03571	0.09968	40	-0.36	0.7220	Dunnett	0.9888	
Group	5	1	0.09726	0.1002	40	0.97	0.3374	Dunnett	0.7276	
