# Supplementary material for: Reproducibility discrepancies following reanalysis of raw data for a previously published study on diisononyl phthalate (DINP) in rats
Source: Data Brief. 2017 May 26;13:208–13. doi: 10.1016/j.dib.2017.05.043 (PMC5459566; doi:10.1016/j.dib.2017.05.043)
Supplement: Supplementary file 3 — Supplementary material [file mmc3.rtf]

Model Information	
Data Set	WORK.TEMP		
Distribution	Poisson		
Link Function	Log		
Dependent Variable	Nipples_male	Nipples-male	


Number of Observations Read	196	
Number of Observations Used	196	


Class Level Information	
Class	Value	Design Variables	
Group	1	0	0	0	0	
	2	1	0	0	0	
	3	0	1	0	0	
	4	0	0	1	0	
	5	0	0	0	1	


Parameter Information	
Parameter	Effect	Group	
Prm1	Intercept		
Prm2	Group	2	
Prm3	Group	3	
Prm4	Group	4	
Prm5	Group	5	


Algorithm converged.	


GEE Model Information	
Correlation Structure	Exchangeable	
Subject Effect	Litter (43 levels)	
Number of Clusters	43	
Correlation Matrix Dimension	8	
Maximum Cluster Size	8	
Minimum Cluster Size	1	


Algorithm converged.	


Working Correlation Matrix	
	Col1	Col2	Col3	Col4	Col5	Col6	Col7	Col8	
Row1	1.0000	0.2172	0.2172	0.2172	0.2172	0.2172	0.2172	0.2172	
Row2	0.2172	1.0000	0.2172	0.2172	0.2172	0.2172	0.2172	0.2172	
Row3	0.2172	0.2172	1.0000	0.2172	0.2172	0.2172	0.2172	0.2172	
Row4	0.2172	0.2172	0.2172	1.0000	0.2172	0.2172	0.2172	0.2172	
Row5	0.2172	0.2172	0.2172	0.2172	1.0000	0.2172	0.2172	0.2172	
Row6	0.2172	0.2172	0.2172	0.2172	0.2172	1.0000	0.2172	0.2172	
Row7	0.2172	0.2172	0.2172	0.2172	0.2172	0.2172	1.0000	0.2172	
Row8	0.2172	0.2172	0.2172	0.2172	0.2172	0.2172	0.2172	1.0000	


Exchangeable Working Correlation	
Correlation	0.2171601828	


GEE Fit Criteria	
QIC	15.1131	
QICu	5.8947	


Analysis Of GEE Parameter Estimates	
Empirical Standard Error Estimates	
Parameter		Estimate	Standard Error	95% Confidence Limits	Z	Pr > |Z|	
Intercept		0.7008	0.1485	0.4097	0.9920	4.72	<.0001	
Group	2	-0.0257	0.1828	-0.3840	0.3326	-0.14	0.8883	
Group	3	0.3556	0.1660	0.0303	0.6809	2.14	0.0321	
Group	4	0.4847	0.1865	0.1192	0.8503	2.60	0.0093	
Group	5	0.4368	0.1716	0.1005	0.7730	2.55	0.0109	

Model Information	
Data Set	WORK.TEMP	
Distribution	Poisson	
Link Function	Log	
Dependent Variable	Nipples_female1	


Number of Observations Read	222	
Number of Observations Used	222	


Class Level Information	
Class	Value	Design Variables	
Group	1	0	0	0	0	
	2	1	0	0	0	
	3	0	1	0	0	
	4	0	0	1	0	
	5	0	0	0	1	


Parameter Information	
Parameter	Effect	Group	
Prm1	Intercept		
Prm2	Group	2	
Prm3	Group	3	
Prm4	Group	4	
Prm5	Group	5	


Algorithm converged.	


GEE Model Information	
Correlation Structure	Exchangeable	
Subject Effect	Litter (45 levels)	
Number of Clusters	45	
Correlation Matrix Dimension	12	
Maximum Cluster Size	12	
Minimum Cluster Size	1	


Algorithm converged.	


Working Correlation Matrix	
	Col1	Col2	Col3	Col4	Col5	Col6	Col7	Col8	Col9	Col10	Col11	Col12	
Row1	1.0000	-0.0465	-0.0465	-0.0465	-0.0465	-0.0465	-0.0465	-0.0465	-0.0465	-0.0465	-0.0465	-0.0465	
Row2	-0.0465	1.0000	-0.0465	-0.0465	-0.0465	-0.0465	-0.0465	-0.0465	-0.0465	-0.0465	-0.0465	-0.0465	
Row3	-0.0465	-0.0465	1.0000	-0.0465	-0.0465	-0.0465	-0.0465	-0.0465	-0.0465	-0.0465	-0.0465	-0.0465	
Row4	-0.0465	-0.0465	-0.0465	1.0000	-0.0465	-0.0465	-0.0465	-0.0465	-0.0465	-0.0465	-0.0465	-0.0465	
Row5	-0.0465	-0.0465	-0.0465	-0.0465	1.0000	-0.0465	-0.0465	-0.0465	-0.0465	-0.0465	-0.0465	-0.0465	
Row6	-0.0465	-0.0465	-0.0465	-0.0465	-0.0465	1.0000	-0.0465	-0.0465	-0.0465	-0.0465	-0.0465	-0.0465	
Row7	-0.0465	-0.0465	-0.0465	-0.0465	-0.0465	-0.0465	1.0000	-0.0465	-0.0465	-0.0465	-0.0465	-0.0465	
Row8	-0.0465	-0.0465	-0.0465	-0.0465	-0.0465	-0.0465	-0.0465	1.0000	-0.0465	-0.0465	-0.0465	-0.0465	
Row9	-0.0465	-0.0465	-0.0465	-0.0465	-0.0465	-0.0465	-0.0465	-0.0465	1.0000	-0.0465	-0.0465	-0.0465	
Row10	-0.0465	-0.0465	-0.0465	-0.0465	-0.0465	-0.0465	-0.0465	-0.0465	-0.0465	1.0000	-0.0465	-0.0465	
Row11	-0.0465	-0.0465	-0.0465	-0.0465	-0.0465	-0.0465	-0.0465	-0.0465	-0.0465	-0.0465	1.0000	-0.0465	
Row12	-0.0465	-0.0465	-0.0465	-0.0465	-0.0465	-0.0465	-0.0465	-0.0465	-0.0465	-0.0465	-0.0465	1.0000	


Exchangeable Working Correlation	
Correlation	-0.046506148	


GEE Fit Criteria	
QIC	-455928.2664	
QICu	-455925.3352	


Analysis Of GEE Parameter Estimates	
Empirical Standard Error Estimates	
Parameter		Estimate	Standard Error	95% Confidence Limits	Z	Pr > |Z|	
Intercept		2.5078	0.0065	2.4951	2.5204	388.10	<.0001	
Group	2	0.0000	0.0077	-0.0150	0.0150	0.00	0.9994	
Group	3	0.0024	0.0084	-0.0140	0.0187	0.28	0.7761	
Group	4	-0.0021	0.0072	-0.0162	0.0120	-0.29	0.7681	
Group	5	0.0085	0.0080	-0.0072	0.0241	1.06	0.2888	
