# Supplementary material for: Reproducibility discrepancies following reanalysis of raw data for a previously published study on diisononyl phthalate (DINP) in rats
Source: Data Brief. 2017 May 26;13:208–13. doi: 10.1016/j.dib.2017.05.043 (PMC5459566; doi:10.1016/j.dib.2017.05.043)
Supplement: Supplementary file 4 — Supplementary material [file mmc4.rtf]

Group=.


Analysis Variable : littermean AG-male	
Mean	Std Dev	Std Error	
.	.	.	


Group=1

Analysis Variable : littermean AG-male	
Mean	Std Dev	Std Error	
21.5035714	1.8178249	0.6870732	


Group=2

Analysis Variable : littermean AG-male	
Mean	Std Dev	Std Error	
21.0949405	1.5816768	0.5592072	


Group=3

Analysis Variable : littermean AG-male	
Mean	Std Dev	Std Error	
20.6543651	2.1112440	0.6676340	


Group=4

Analysis Variable : littermean AG-male	
Mean	Std Dev	Std Error	
20.6666667	1.5500896	0.5480394	


Group=5

Analysis Variable : littermean AG-male	
Mean	Std Dev	Std Error	
19.8226190	1.4140738	0.4471694	

Group=.


Analysis Variable : littermean AGI male	
Mean	Std Dev	Std Error	
.	.	.	


Group=1

Analysis Variable : littermean AGI male	
Mean	Std Dev	Std Error	
11.5973545	1.0383433	0.3924569	


Group=2

Analysis Variable : littermean AGI male	
Mean	Std Dev	Std Error	
11.4296137	0.8217176	0.2905210	


Group=3

Analysis Variable : littermean AGI male	
Mean	Std Dev	Std Error	
11.3068163	1.1990306	0.3791668	


Group=4

Analysis Variable : littermean AGI male	
Mean	Std Dev	Std Error	
11.2929908	0.7489617	0.2647980	


Group=5

Analysis Variable : littermean AGI male	
Mean	Std Dev	Std Error	
10.9363021	0.8616427	0.2724754	

Group=.


Analysis Variable : littermean Nipples-male	
Mean	Std Dev	Std Error	
.	.	.	


Group=1

Analysis Variable : littermean Nipples-male	
Mean	Std Dev	Std Error	
1.9773810	0.8299196	0.3136801	


Group=2

Analysis Variable : littermean Nipples-male	
Mean	Std Dev	Std Error	
2.0014881	0.6416175	0.2268461	


Group=3

Analysis Variable : littermean Nipples-male	
Mean	Std Dev	Std Error	
2.9120238	0.6931994	0.2192089	


Group=4

Analysis Variable : littermean Nipples-male	
Mean	Std Dev	Std Error	
3.1354167	1.2149348	0.4295443	


Group=5

Analysis Variable : littermean Nipples-male	
Mean	Std Dev	Std Error	
3.2154762	0.8908788	0.2817206	

Group=.


Analysis Variable : littermean Birth-bw-male	
Mean	Std Dev	Std Error	
.	.	.	


Group=1

Analysis Variable : littermean Birth-bw-male	
Mean	Std Dev	Std Error	
6.3911905	0.3873064	0.1463880	


Group=2

Analysis Variable : littermean Birth-bw-male	
Mean	Std Dev	Std Error	
6.2954762	0.1235932	0.0436968	


Group=3

Analysis Variable : littermean Birth-bw-male	
Mean	Std Dev	Std Error	
6.1218730	0.3156546	0.0998187	


Group=4

Analysis Variable : littermean Birth-bw-male	
Mean	Std Dev	Std Error	
6.1406250	0.4867579	0.1720949	


Group=5

Analysis Variable : littermean Birth-bw-male	
Mean	Std Dev	Std Error	
5.9901190	0.5422654	0.1714794	

Group=.


Analysis Variable : littermean	
Mean	Std Dev	Std Error	
.	.	.	


Group=1

Analysis Variable : littermean	
Mean	Std Dev	Std Error	
11.0531250	0.8980369	0.3175040	


Group=2

Analysis Variable : littermean	
Mean	Std Dev	Std Error	
11.3231481	0.8527710	0.2842570	


Group=3

Analysis Variable : littermean	
Mean	Std Dev	Std Error	
11.3761905	1.2962434	0.4099081	


Group=4

Analysis Variable : littermean	
Mean	Std Dev	Std Error	
11.2735450	0.8562830	0.2854277	


Group=5

Analysis Variable : littermean	
Mean	Std Dev	Std Error	
10.7039683	0.6584054	0.2194685	

Group=.


Analysis Variable : littermean	
Mean	Std Dev	Std Error	
.	.	.	


Group=1

Analysis Variable : littermean	
Mean	Std Dev	Std Error	
6.0962529	0.4966636	0.1755971	


Group=2

Analysis Variable : littermean	
Mean	Std Dev	Std Error	
6.2722258	0.4778162	0.1592721	


Group=3

Analysis Variable : littermean	
Mean	Std Dev	Std Error	
6.2592091	0.6742317	0.2132108	


Group=4

Analysis Variable : littermean	
Mean	Std Dev	Std Error	
6.2473171	0.3849117	0.1283039	


Group=5

Analysis Variable : littermean	
Mean	Std Dev	Std Error	
5.9776926	0.4145483	0.1381828	

Group=.


Analysis Variable : littermean	
Mean	Std Dev	Std Error	
.	.	.	


Group=1

Analysis Variable : littermean	
Mean	Std Dev	Std Error	
12.2986607	0.2615965	0.0924883	


Group=2

Analysis Variable : littermean	
Mean	Std Dev	Std Error	
12.2481481	0.1877211	0.0625737	


Group=3

Analysis Variable : littermean	
Mean	Std Dev	Std Error	
12.2970238	0.2416150	0.0764054	


Group=4

Analysis Variable : littermean	
Mean	Std Dev	Std Error	
12.2359788	0.1440252	0.0480084	


Group=5

Analysis Variable : littermean	
Mean	Std Dev	Std Error	
12.3388889	0.2490686	0.0830229	

Group=.


Analysis Variable : littermean	
Mean	Std Dev	Std Error	
.	.	.	


Group=1

Analysis Variable : littermean	
Mean	Std Dev	Std Error	
5.9692857	0.3093321	0.1093654	


Group=2

Analysis Variable : littermean	
Mean	Std Dev	Std Error	
5.8954630	0.1866477	0.0622159	


Group=3

Analysis Variable : littermean	
Mean	Std Dev	Std Error	
6.0084524	0.3206715	0.1014052	


Group=4

Analysis Variable : littermean	
Mean	Std Dev	Std Error	
5.8806349	0.4137981	0.1379327	


Group=5

Analysis Variable : littermean	
Mean	Std Dev	Std Error	
5.7606349	0.2906194	0.0968731	

Group=Control


Analysis Variable : littermean	
Mean	Std Dev	Std Error	
59.3055556	5.5561944	2.2683069	


Group=DINP 300

Analysis Variable : littermean	
Mean	Std Dev	Std Error	
57.1428571	5.7085288	2.1576211	


Group=DINP 600

Analysis Variable : littermean	
Mean	Std Dev	Std Error	
51.7000000	6.0973583	1.9281540	


Group=DINP 750

Analysis Variable : littermean	
Mean	Std Dev	Std Error	
48.2142857	7.6477510	2.8905782	


Group=DINP 900

Analysis Variable : littermean	
Mean	Std Dev	Std Error	
47.3571429	5.7978513	2.1913818	

Group=Control


Analysis Variable : littermean	
Mean	Std Dev	Std Error	
31.9444444	6.6954932	2.7334237	


Group=DINP 300

Analysis Variable : littermean	
Mean	Std Dev	Std Error	
33.3095238	4.3209530	1.6331667	


Group=DINP 600

Analysis Variable : littermean	
Mean	Std Dev	Std Error	
28.7500000	6.8647651	2.1708293	


Group=DINP 750

Analysis Variable : littermean	
Mean	Std Dev	Std Error	
25.0238095	5.7473251	2.1722847	


Group=DINP 900

Analysis Variable : littermean	
Mean	Std Dev	Std Error	
26.1666667	3.9732905	1.5017626	

Group=Control


Analysis Variable : littermean Sperm/g	
Mean	Std Dev	Std Error	
421.0805556	68.8707779	28.1163773	


Group=DINP 300

Analysis Variable : littermean Sperm/g	
Mean	Std Dev	Std Error	
482.6523810	37.4911439	14.1703205	


Group=DINP 600

Analysis Variable : littermean Sperm/g	
Mean	Std Dev	Std Error	
460.4100000	52.2608777	16.5263406	


Group=DINP 750

Analysis Variable : littermean Sperm/g	
Mean	Std Dev	Std Error	
431.5595238	49.1650403	18.5826386	


Group=DINP 900

Analysis Variable : littermean Sperm/g	
Mean	Std Dev	Std Error	
497.8261905	13.5080218	5.1055523	

Group=Control


Analysis Variable : littermean	
Mean	Std Dev	Std Error	
228.7777778	11.1230275	4.5409570	


Group=DINP 300

Analysis Variable : littermean	
Mean	Std Dev	Std Error	
214.4619048	17.5024805	6.6153158	


Group=DINP 600

Analysis Variable : littermean	
Mean	Std Dev	Std Error	
226.1550000	21.7205158	6.8686302	


Group=DINP 750

Analysis Variable : littermean	
Mean	Std Dev	Std Error	
212.0190476	21.5973773	8.1630413	


Group=DINP 900

Analysis Variable : littermean	
Mean	Std Dev	Std Error	
211.9952381	9.9448931	3.7588163	

Group=Control


Analysis Variable : littermean sperm count	
Mean	Std Dev	Std Error	
98.0917011	15.7693542	6.4378119	


Group=DINP 300

Analysis Variable : littermean sperm count	
Mean	Std Dev	Std Error	
103.6428529	14.6641621	5.5425323	


Group=DINP 600

Analysis Variable : littermean sperm count	
Mean	Std Dev	Std Error	
103.4980590	13.5093511	4.2720319	


Group=DINP 750

Analysis Variable : littermean sperm count	
Mean	Std Dev	Std Error	
92.2670890	13.3228201	5.0355527	


Group=DINP 900

Analysis Variable : littermean sperm count	
Mean	Std Dev	Std Error	
105.5832917	5.1654094	1.9523412	

group=1


Analysis Variable : littermean ng per testis	
Mean	Std Dev	Std Error	
8.7864000	1.5136677	0.8739164	


group=2

Analysis Variable : littermean ng per testis	
Mean	Std Dev	Std Error	
7.6140000	0.8453487	0.4226743	


group=3

Analysis Variable : littermean ng per testis	
Mean	Std Dev	Std Error	
4.3786800	2.4775320	1.2387660	


group=4

Analysis Variable : littermean ng per testis	
Mean	Std Dev	Std Error	
6.1128000	2.6869316	1.5513007	


group=5

Analysis Variable : littermean ng per testis	
Mean	Std Dev	Std Error	
5.4768000	0.4532189	0.2616660	

group=1


Analysis Variable : littermean Testicular testosterone production	
Mean	Std Dev	Std Error	
2.5066667	2.3474525	1.3553023	


group=2

Analysis Variable : littermean Testicular testosterone production	
Mean	Std Dev	Std Error	
1.2162500	0.4194714	0.2097357	


group=3

Analysis Variable : littermean Testicular testosterone production	
Mean	Std Dev	Std Error	
0.6425000	0.3657071	0.1828535	


group=4

Analysis Variable : littermean Testicular testosterone production	
Mean	Std Dev	Std Error	
0.7850000	0.6064102	0.3032051	


group=5

Analysis Variable : littermean Testicular testosterone production	
Mean	Std Dev	Std Error	
0.5962500	0.4240553	0.2120277	

Group=Control


Analysis Variable : littermean	
Mean	Std Dev	Std Error	
164.4444444	7.5929475	3.0998078	


Group=DINP 300

Analysis Variable : littermean	
Mean	Std Dev	Std Error	
161.3571429	13.0284264	4.9242823	


Group=DINP 600

Analysis Variable : littermean	
Mean	Std Dev	Std Error	
163.9750000	9.9246956	3.1384643	


Group=DINP 750

Analysis Variable : littermean	
Mean	Std Dev	Std Error	
155.3666667	7.0977892	2.6827121	


Group=DINP 900

Analysis Variable : littermean	
Mean	Std Dev	Std Error	
163.5000000	14.8202689	5.6015351	

Group=Control


Analysis Variable : littermean	
Mean	Std Dev	Std Error	
331.1305556	22.1687679	9.0503616	


Group=DINP 300

Analysis Variable : littermean	
Mean	Std Dev	Std Error	
332.8404762	22.1976264	8.3899142	


Group=DINP 600

Analysis Variable : littermean	
Mean	Std Dev	Std Error	
333.1200000	22.4682269	7.1050772	


Group=DINP 750

Analysis Variable : littermean	
Mean	Std Dev	Std Error	
324.4761905	10.5943812	4.0042997	


Group=DINP 900

Analysis Variable : littermean	
Mean	Std Dev	Std Error	
346.2571429	29.4660615	11.1371244	

Group=Control


Analysis Variable : littermean	
Mean	Std Dev	Std Error	
98.8166667	6.0568786	2.4727103	


Group=DINP 300

Analysis Variable : littermean	
Mean	Std Dev	Std Error	
97.6142857	9.3013511	3.5155803	


Group=DINP 600

Analysis Variable : littermean	
Mean	Std Dev	Std Error	
99.1400000	9.4366249	2.9841228	


Group=DINP 750

Analysis Variable : littermean	
Mean	Std Dev	Std Error	
94.5095238	7.7481052	2.9285085	


Group=DINP 900

Analysis Variable : littermean	
Mean	Std Dev	Std Error	
96.3285714	13.3465966	5.0445393	

Group=Control


Analysis Variable : littermean STR	
Mean	Std Dev	Std Error	
60.1666667	1.7857460	0.7290278	


Group=DINP 300

Analysis Variable : littermean STR	
Mean	Std Dev	Std Error	
60.5714286	1.5952973	0.6029657	


Group=DINP 600

Analysis Variable : littermean STR	
Mean	Std Dev	Std Error	
60.7000000	2.7808871	0.8793937	


Group=DINP 750

Analysis Variable : littermean STR	
Mean	Std Dev	Std Error	
60.7142857	3.3398745	1.2623539	


Group=DINP 900

Analysis Variable : littermean STR	
Mean	Std Dev	Std Error	
59.6666667	2.8218722	1.0665675	
