# Supplementary material for: Reproducibility discrepancies following reanalysis of raw data for a previously published study on diisononyl phthalate (DINP) in rats
Source: Data Brief. 2017 May 26;13:208–13. doi: 10.1016/j.dib.2017.05.043 (PMC5459566; doi:10.1016/j.dib.2017.05.043)
Supplement: Supplementary file 6 — Supplementary material [file mmc6.docx]

/*litter as an independent, random and nested factor in ANOVA, Dunnett's test was performed to determine differences between treated and control group means*/

**%macro** mixed(data,y,x);

proc mixed data=&data;

class litter group;

model &y=group &x/s;

random litter(group)/ cl alpha=**0.05**;

lsmeans group/adjust=dunnett;

run;

**%mend**;

/*****calculate mean standard error and standard deviation******/

**%macro** mean(data,var);

proc sort data=&data;by litter group;run;

proc means data=&data noprint;

var &var;

by litter group;

output out=a mean=littermean;

run;

proc sort data=a; by group;run;

proc means data=a mean STD STDERR ;

var littermean;

by group;

run;

**%mend**;

/***GLM in combination with generalized estimating equations in order

to account for the nested litter correlation analyzed the number of nipples***/

title 'Nipples male';

**Proc** **genmod** data=temp ;

where nipples_male ne **.**;

Class litter group(ref="1")/param=ref ;

Model nipples_male=group/dist=poisson ;

repeated subject=litter / type=exch corrw;

**run**;

title 'Nipples female';

**Proc** **genmod** data=temp ;

where nipples_female ne **.**;

Class litter group(ref="1")/param=ref ;

Model nipples_female=group/dist=poisson;

repeated subject=litter / type=exch corrw;

**run**;
